# Optimizing Technology-Based Prompts for Supporting People Living With Dementia in Completing Activities of Daily Living at Home: Experimental Approach to Prompt Modality, Task Breakdown, and Attentional Support

**DOI:** 10.2196/56055

**Published:** 2024-08-23

**Authors:** Madeleine Cannings, Ruth Brookman, Simon Parker, Leonard Hoon, Asuka Ono, Hiroaki Kawata, Hisashi Matsukawa, Celia B Harris

**Affiliations:** 1 The MARCS Institute for Brain, Behaviour, and Development Western Sydney University Penrith Australia; 2 Applied Artificial Intelligence Institute Deakin University Burwood Australia; 3 Nippon Telegraph and Telephone Tokyo Japan

**Keywords:** assistive technology, accessible technology, accessibility technology, assistive technologies, accessible technologies, assistive device, assistive devices, dementia, people living with dementia, dementia care, person-centered technology, patient-centered technology, person-centered technologies, patient-centered technologies, memory support, prompting, user-computer interface, user interface, UI, app, apps, digital health, digital technology, digital intervention, digital interventions, mobile phone

## Abstract

**Background:**

Assistive technology is becoming increasingly accessible and affordable for supporting people with dementia and their care partners living at home, with strong potential for technology-based prompting to assist with initiation and tracking of complex, multistep activities of daily living. However, there is limited direct comparison of different prompt features to guide optimal technology design.

**Objective:**

Across 3 experiments, we investigated the features of tablet-based prompts that best support people with dementia to complete activities of daily living at home, measuring prompt effectiveness and gaining feedback from people with dementia and their care partners about their experiences.

**Methods:**

Across experiments, we developed a specialized iPad app to enable data collection with people with dementia at home over an extended experimental period. In experiment 1, we varied the prompts in a 3 (visual type: text instruction, iconic image, and photographic image) × 3 (audio type: no sound, symbolic sound, and verbal instruction) experimental design using repeated measures across multiple testing sessions involving single-step activities. In experiment 2, we tested the most effective prompt breakdown for complex multistep tasks comparing 3 conditions (1-prompt, 3-prompt, and 7-prompt conditions). In experiment 3, we compared initiation and maintenance alerts that involved either an auditory tone or an auditory tone combined with a verbal instruction. Throughout, we asked people with dementia and their care partners to reflect on the usefulness of prompting technology in their everyday lives and what could be developed to better meet their needs.

**Results:**

First, our results showed that audible verbal instructions were more useful for task completion than either tone-based or visual prompts. Second, a more granular breakdown of tasks was generally more useful and increased independent use, but this varied across individuals. Third, while a voice or text maintenance alert enabled people with dementia to persist with a multistep task for longer when it was more frequent, task initiation still frequently required support from a care partner.

**Conclusions:**

These findings can help inform developers of assistive technology about the design features that promote the usefulness of home prompting systems for people with dementia as well as the preferences and insights of people with dementia and their care partners regarding assistive technology design.

## Introduction

### Background

Dementia is a progressive neurological condition that results in a range of cognitive and motor impairments that can have an impact on an individual’s well-being and capacity to live independently. These impacts include difficulties with orientation in time and space; noticing body signals such as hunger, thirst, or temperature; initiating activities; and keeping track of a sequence of tasks [1]. Assistive technology that provides prompts for activities of daily living (ADLs) is a form of environmental support that can help people living with dementia by reducing the impact of their cognitive impairment and enabling active living and independence [2]. In daily practical terms, a prompting device could be an effective way to support people with dementia to first notice that an ADL needs to be done; then initiate the ADL; and, finally, undertake each step of the ADL in the necessary sequence to see it through to completion. In this way, assistive technology has strong potential to support people with dementia compensate for their cognitive disability. However, the cognitive, sensory, and motor changes associated with aging and dementia may have an impact on design features of technology interfaces that are useful for this user group [3]. So far, little research has directly compared the outcomes of different types of prompts (eg, visual vs auditory) to test what is most useful for people with dementia. Across 3 experiments, we addressed this gap, with the aim of informing general assistive technology design for people with dementia.

ADLs are functional, multistep, complex tasks related to self-care, domestic duties, socialization, and leisure. Difficulties with completing ADLs can arise for a range of reasons, and these difficulties have a substantial impact on quality of life for people with dementia. People with dementia often experience reduced ability to initiate activities as well as executive functioning changes that make accurate tracking of multistep activities challenging [4,5]. These difficulties with ADLs increase dependence on others for support [6] and are a common reason for moving to residential care [7]. Supporting people with dementia to both initiate activities and accurately and independently complete the steps involved in daily tasks has the potential to improve well-being for both people with dementia and their family care partners [6,8]. Therefore, cognitive rehabilitation approaches emphasize the potential for the right support tools and strategies to enable successful ADL completion and support people with dementia in living independently for longer [9].

Assistive technology has increasingly received attention for its potential to reduce the impact of cognitive impairments on an individual’s day-to-day functioning [10]. However, existing research on prompting technology for people with dementia has adopted a wide range of approaches, which makes drawing general conclusions and developing recommendations challenging. Technology solutions vary in several ways, including what kinds of tasks they aim to support, what kind of device they adopt, prompt features, and the amount of user involvement required. Typically, a single version of the prompting system is developed and then user tested, often involving small feasibility trials [11]. Studies that directly compare design features in terms of their outcomes for people with dementia are scarce [11]. More broadly, technological research and development at the cutting edge of innovation has to date been unable to generate solutions that are readily available on the market. One reason is that people with dementia and family care partners experience barriers and challenges in incorporating assistive technology into daily care practice, contributing to the low uptake of existing devices [12-14]. Therefore, experimental work and an evidence base with end users is required to inform general technology design for people with dementia to ensure that assistive technology solutions go beyond research studies and become part of everyday life [15,16].

### Device Types and User Involvement

A recent review identified 30 published studies of prompting systems to support people with a cognitive impairment in completing ADLs [11]. The authors classified 6 broad types of devices, with varying hardware approaches and corresponding variance in the extent to which users were involved in mediating prompt delivery. Technology solutions ranged from fully autonomous sensor-based home systems designed to detect completed steps in a sequence and provide prompts when needed to social robots and prompts delivered in readily available consumer devices such as tablets and smartphones. Systems that are autonomous, without user input, typically use sensors and smart technology to monitor a person’s activity and detect where they are up to in a sequence of steps [17] to determine when a prompt may need to be given. Examples of these systems tend to focus on supporting a specific ADL for which the system is designed and trained, such as the Cognitive Orthosis for Assisting Activities in the Home (COACH) system for handwashing [18]. Alternative approaches involve input from either people with dementia or care partners to determine when tasks are initiated and when prompts are delivered. Examples of these include app-based prompting software where the user themselves selects *next* to move through steps and receive the next prompt in a sequence [19], although these solutions may not align with people with dementia’s need for support with activity initiation. Interestingly, in a recent version of the COACH handwashing system [20], researchers reported that voice-based interaction with the system was much more effective for users with dementia than the previous iterations that had used a camera to detect step completion, indicating that a human-in-the-loop system may be more effective than a fully automated one. Across these varying device types and technologies—from apps to sensors to social robots—the features of effective prompts have not yet been taxonomized. We aimed to test general principles about the optimal design features of effective prompts for people with dementia in ways that could be applied to these widely ranging device types, technologies, and intended tasks.

### Optimizing Prompt Design: Modality, Timing, and Breakdown

Existing research provides a limited evidence base on how to optimize prompts for people with dementia in terms of features such as modality, timing, and task breakdown. Prompting technologies in the published literature have adopted a range of modalities for prompt delivery, including a visual (eg, image- or video-based instruction) or auditory (eg, verbal instruction [21]) element or a combination of both [22,23]. Prompt modality has been frequently determined by device design, although some researchers have provided the individual user with a choice [12,24]. In the subset of studies in which outcomes have been measured and reported, studies that have compared a *prompting* to a *no prompting* condition generally have demonstrated some advantage to using prompts [11]. For example, a recent experimental study tested a smartphone app that prompted water drinking and found significantly better performance in the prompted than in the unprompted condition [25]. Reviews have identified benefits of step-based prompting in other populations (eg, those with an intellectual disability [26]). The social robot Tessa has been used to provide people with dementia with support to complete a predetermined ADL goal [27]. The eWare system integrates the social robot Tessa with sensor monitoring technology (eg, door contact sensors) with the aim of using the sensor data to generate verbal reminders and motivating comments for people with dementia. A small pilot study of 9 dyads who were monitored over 6 months [28] found promising results of this system but with limited benefits for carer burden and the need for further research identified.

Despite promising evidence that technology-based prompting can support ADL completion for people with dementia, few studies have directly compared different prompt modalities, timings, and task breakdown options to determine which design features are the most useful and direct future technology development. In one exception, a study directly comparing prompt features found that text-based and verbal prompts were more effective than image-based prompts for a card-and-envelope task but not for putting on a CD to play music [19]. The authors concluded that the most effective prompt modality may depend on aspects of the task, including complexity and familiarity (see also the study by Braley et al [22]). In a qualitative study with the social robot Tessa, people with a cognitive disability and their carers preferred prompts with an auditory element compared to visual-only prompts [29]. Conversely, other research has suggested no advantage in adding verbal instructions to visual (pictorial or video) prompts, albeit with small sample sizes [30,31]. Overall, although there is some evidence that the presence of prompts is better than no prompts, there are limited data to guide choices about optimal prompt design, including prompt modality, task breakdown, and timing.

### Task Types and Task Breakdown

Within the literature, a number of prompting systems have been developed that are specialized to support 1 key activity of ranging complexity, including washing hands [32,33], putting on prosthetic limbs [34], making pizza [35], morning routine [36], and cooking tasks [37]. Some prompting systems could theoretically be applied to any task but were tested with a small range of predetermined tasks for the purposes of evaluation [23,25,30,31]. Other devices have focused on a generic interface that can support any kind of user-chosen task. For instance, research that examined a personalized tablet-based prompter found that people with dementia and their care partners could select and load their own tasks and steps based on their own goals [24]. Tasks chosen by users included using the television or a camera, remembering to take required objects when leaving the house, and basic ADLs such as table setting or making a sandwich [24]. In our own research conducting detailed interviews with people with dementia and their care partners, we identified a great deal of individual variation in what tasks were valued by individuals as well as idiosyncratic reasons for task incompletion and which steps within tasks needed more support [38]. Despite the technological challenges of an open-ended system in which people can choose their own tasks and prompts, this personalized approach appears to more closely match the needs of users within their everyday context.

### This Research

A number of recent studies of home prompting systems for people with dementia have focused on implementing prompts via mainstream, tablet-based devices. Research indicates good potential for the usability of these systems such that people with dementia and their care partners can interact with a touch-screen tablet by, for example, advancing to the next step in a sequence via pressing an on-screen button [19]. However, a recent review concluded that general conclusions could not be drawn about the effectiveness of prompting technology for people with dementia because many studies were very small and did not have an experimental design with comparisons and control groups [11].

To address this gap, we conducted a series of 3 iterative studies aiming to advance assistive technology design by examining the potential of prompts to support people with dementia with 2 key aspects of ADL completion. First, people with dementia often experience loss of initiative and could benefit from technology that activates them to commence a task through effective reminders. Second, people with dementia experience difficulties with executive functioning, making multistep activities more difficult to perform and keep track of, so that complex tasks may need multiple prompts provided in a sequence to be completed successfully. The 3 studies integrated experimental design and both quantitative and qualitative methods and examined prompt modality (experiment 1), task breakdown (experiment 2), task initiation (experiment 3), and attention maintenance (experiment 3). At the conclusion of each study, we gained detailed user experiences and feedback to contextualize our findings and provide user recommendations for future technology development.

### Ethical Considerations

All studies were conducted with approval from the Western Sydney University Human Research Ethics Committee (reference H14632). All participants provided extended consent for both primary data collection and secondary analyses of the research data. Additional proxy consent was obtained from family members in situations in which participants with dementia—through cognitive screening or specialist advice—were assessed to have reduced capacity to provide informed consent depending on their degree of cognitive impairment. In all three experiments, both participants with dementia and care partners were reimbursed (Aus $50 [US $33.74] per hour e-gift card) in recognition of their time and specialist expertise due to their lived experience of dementia. All methods were carried out in accordance with relevant guidelines and regulations. Data were deidentified for analyses and stored securely on Western Sydney University infrastructure.

### Experiment 1

#### Methods

##### Participants

Participants were 11 people with dementia, 10 (91%) of whom were supported by a care partner to complete the research. Participants with dementia (n=5, 45% female; n=6, 55% male) ranged in age from 58 to 82 years, with an average age of 73 (SD 7.20) years. Participants who were care partners (10 female) ranged in age from 34 to 77 years, with an average age of 58 (SD 13.59) years. A total of 55% (6/11) of the participants with dementia spoke English as a first language, and 45% (5/11) spoke a language other than English. Participants with dementia had been diagnosed between 9 months and 9 years before the study, with an average of 3.5 years. The relationship of care partners to the participants with dementia included 50% (5/10) spouses, 40% (4/10) adult children, and 10% (1/10) siblings. Participants were recruited through care partner support groups, Dementia Australia, and the StepUp for Dementia Research participant database [39]. We did not formally record additional medical information regarding comorbidities beyond the participants’ dementia diagnosis, but participants were required to have sufficient visual and hearing ability to be able to see and hear the iPad prompts as part of our inclusion criteria.

##### Design and Materials

###### Software for Prompt Delivery

We developed a method for remote data collection to accommodate restrictions on face-to-face research associated with the COVID-19 pandemic. A dedicated iPad app was developed to enable data collection in participants’ homes. An iPad was issued to each participant, with reliance on collaboration between researchers and care partners who facilitated data collection with participants with dementia. Using the iPad app, we established 9 conditions to test what visual and auditory features would make prompts most useful. We varied prompts in a 3 (visual content: text instruction, iconic image, and photographic image)×3 (audio content: no sound, symbolic sound, and verbal instruction) experimental design. We also included 5 different tasks developed to sample a range of daily activities that a seated participant could complete with a single action (ie, not involving moving around the home). These tasks were (1) *drink some water*, (2) *brush your teeth*, (3) *wear a mask*, (4) *put on something warm*, and (5) *turn on the television*. The 5 different tasks were completed in each of the 9 conditions, resulting in 45 trials for each participant.

###### Visual Prompt Content

**Figure 1:**
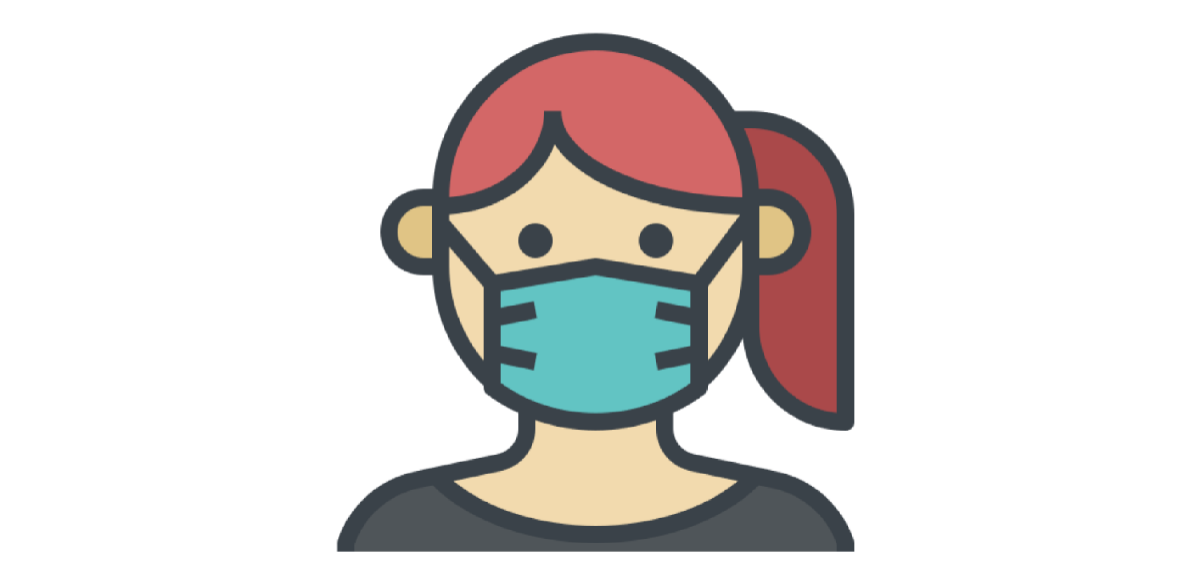
Example item—the icon image for “Wear a mask.”.

For the visual prompts, photo images representing each task were sourced from Unsplash (Getty Images [40]), and icon images representing each task were sourced from Flat Icon (Freepik Company SL; Figure 1).

###### Auditory Prompt Content

For the auditory content, symbolic sounds were developed and recorded by the research team. Verbal instructions were recorded in an Australian-accented female voice as high quality, clearly presented audio clips.

###### Pilot-Testing

The associations among the images, sounds, and the target tasks were pilot-tested by conducting a survey of 55 Australian older adults (aged ≥65 years). We used the responses of these participants to refine our final stimuli selection by presenting a variety of stimuli and selecting the images and sounds rated as having the strongest association with the target task.

##### Measures and Scoring

###### Prompt Usefulness

**Figure 2:**
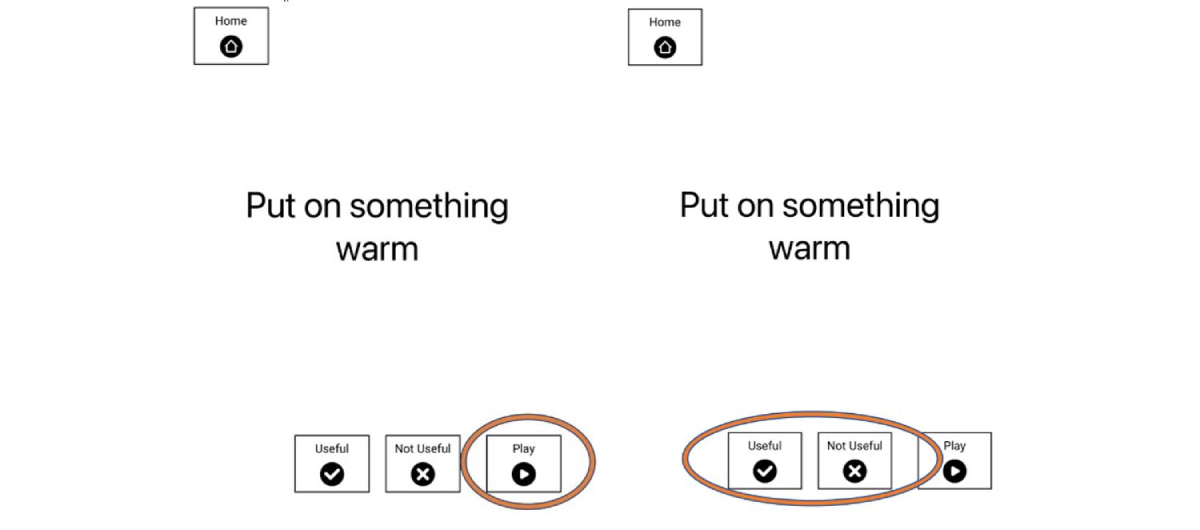
An example item for “Put on something warm” in the text and verbal instruction condition. The prompt screen included the option to press “play” to repeat the auditory content (either symbolic sound or verbal instruction). Carers rated the usefulness of each prompt by pressing the appropriate button.

Our key dependent variable was prompt usefulness. For each prompt, immediately following its presentation, care partners rated whether it was *useful* or *not useful* by selecting the appropriate button on the iPad (Figure 2). For a scoring of useful carers were instructed that this should be selected if the participant with dementia completed or approximated the target task (ie, initiated the task by selecting the correct target item from the table and then followed through by completing the prompted action with it). When tasks were not completed or the prompt could not be understood by the participant with dementia, carers were instructed to rate the prompt as not useful (Figure 2).

###### Time to Respond

The time between prompt onset and the care partner indicating whether it was useful or not useful was recorded automatically via the iPad app. Response times were then compared across prompt conditions.

###### Care Partner Support

To understand the extent to which care partners were involved in supporting the participant with dementia in engaging with the prompts and completing tasks, the iPad app captured audio and video during each trial using the in-built camera. Independent coders scored the videos for verbal assistance from care partners at 3 time points (using the iPad, engaging with the objects on the table, and performing the action) and across 2 levels of assistance (*asking for attention* or *direct telling*). All videos were scored for the presence of support in the resulting 6 categories by 2 raters who were blind to the prompting condition. Agreement between raters was substantial (84.92%), and the ratings of the first rater were retained for analysis.

##### Procedure

###### Baseline

During an initial video call, participants were oriented as to the project. They provided informed consent and proxy consent where appropriate. If participants with dementia had not had a recent cognitive assessment with their health care provider, a Mini-Mental State Examination (MMSE) [41] was conducted to assess the cognitive status of participants with dementia and determine the consent pathway. Participants provided demographics, including age, gender, and cultural and language background, and information about their dementia diagnosis, including when it was received, by whom, and what type of dementia they were diagnosed with (if known).

**Figure 3:**
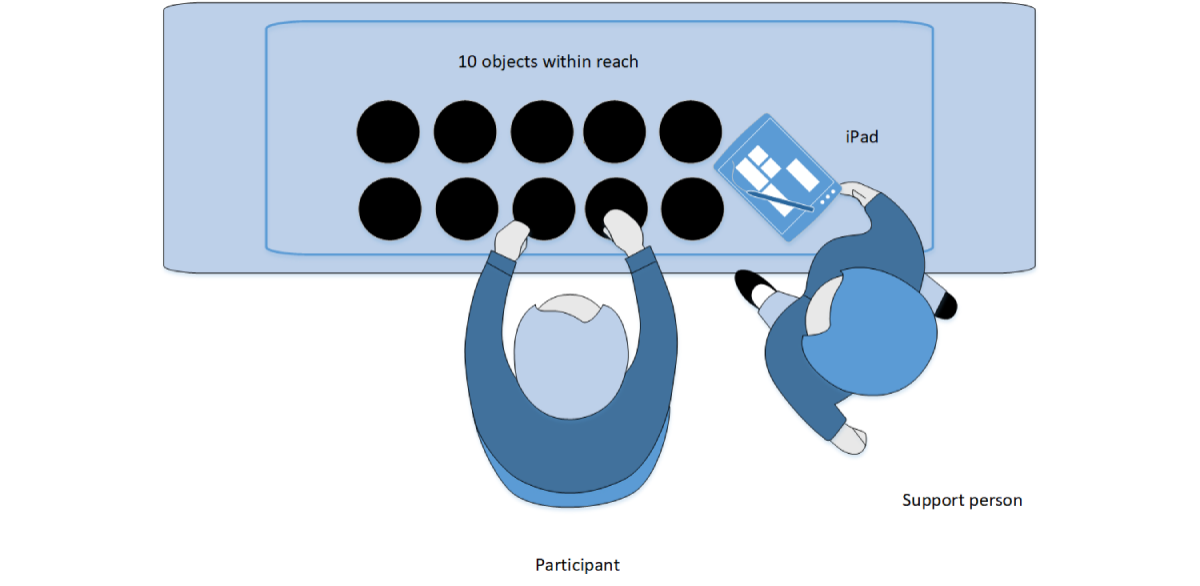
A visual representation of the at-home setup provided within the care partner manual, including positioning of the objects and iPad.

Participants were given a detailed printed manual as well as verbal phone instructions about how to set up and conduct the tasks. Care partners were instructed that participants with dementia should sit comfortably at a table with 10 preselected objects in front of them within easy reach. Objects included 5 target objects (a glass of water, toothbrush, mask, an item of warm clothing like a scarf or woolen hat, and a television remote control) and 5 distractor objects (other familiar objects from around the home). The iPad was placed to the side of the participant with dementia to enable them to see and interact with it while being able to reach the objects on the table (Figure 3).

###### Experimental Phase

On each research participation day, care partners supported participants with dementia to work through a set of 5 prompts for the 5 target tasks. Each of the 5 task types appeared once each day in a different condition, with the condition associated with each task varying in a fixed random order. Across the whole experiment, each of the 5 tasks appeared in each of the 9 conditions, and conditions were spread across the 45 trials (Table 1). For each prompt, participants with dementia were to respond by selecting the appropriate object from in front of them and completing the task. There was a button labelled *play* that enabled care partners and participants with dementia to replay the symbolic sound or verbal instruction if they wished to have it repeated (Figure 2). Care partners were asked to allow participants with dementia to respond to the prompt themselves without assistance. For each prompt, care partners rated whether it was useful (the participant with dementia completed or approximated the task) or not useful (the participant with dementia did not complete the task) by selecting the appropriate button on the iPad (Figure 2). Once rated, the next task was presented on the iPad until the 5 tasks for that day were completed. This procedure was repeated over 9 separate sessions (typically one session each day for consecutive days) until all 45 trials were completed. The iPad automatically captured video and audio during the experimental sessions while the prompts were on the screen using the front-facing iPad camera. In the case of partial completion of the 5 tasks on a particular day, participants and care partners continued the tasks at their next session.

**Table 1:** The 9 experimental conditions for each of the 5 activity of daily living tasks^a^.

Audio condition	Visual condition		
	Text instruction	Iconic image	Photographic image
No sound	Tasks 1-5	Tasks 1-5	Tasks 1-5
Symbolic sound	Tasks 1-5	Tasks 1-5	Tasks 1-5
Verbal instruction	Tasks 1-5	Tasks 1-5	Tasks 1-5

^a^Task 1: “Have a drink of water”; task 2: “Brush your teeth”; task 3: “Wear a mask”; task 4: “Put on something warm”; task 5: “Turn on the television.”

###### Experience and Feedback Interview

After completion of the 9-day experimental phase, a semistructured interview was conducted with both the participant with dementia and the care partner together. Questions included the following: “How did you and the person you care for engage with the system? What worked and what didn’t work?”; “What did you think of the images, sounds, and text?”; and “What would you use a system like this for in your daily life?” The interviews were approximately 15 to 20 minutes in duration. Due to restrictions on face-to-face interactions with participants, all interviews were conducted via web-based videoconference and recorded for transcription and analysis. The transcripts were checked and edited for accuracy. Transcripts were stored and analyzed by the research team in the NVivo qualitative analysis software (QSR International) using a coding rubric as a guide to code. A thematic approach was taken to identify, analyze, and interpret patterns of meaning [42].

#### Results

##### Prompt Usefulness

To determine what kinds of prompts were most useful, we first examined the frequency with which care partners responded *useful* versus *not useful* for each task type. A chi-square analysis indicated that prompt usefulness was significantly different across task types (*χ*^2^_4_=35.3; *P*<.001), with “drink some water” (79/100 trials, 79%), “brush your teeth” (80/100 trials, 80%), and “put on a mask” (82/100 trials, 82%) being more successful than “turn on the TV” (53/100 trials, 53%) and “put on something warm” (59/100 trials, 59%). These differences in usefulness may be due to the direct correspondence between the stimuli and the item in front of the participants with dementia that they were to select. For example, compared with the other target objects (glass, toothbrush, and mask), the link between the target objects for “turn on the TV” (television remote control) and “put on something warm” (item of clothing) was more abstract, without a direct correspondence between the visual prompt (eg, a television) and the item on the table (eg, the television remote control).

Participants varied in their stage of dementia and degree of cognitive impairment. Therefore, we examined any differences in prompt usefulness depending on dementia severity, which was self-reported by care partners as mild, mild to moderate, moderate, or severe. Self-reporting was also corroborated, where possible, through administration of the MMSE [41]. Data indicated a difference in prompt usefulness depending on the participants’ dementia severity (*χ*^2^_3_=61.9; *P*<.001). Frequencies of prompts being rated as useful were comparable for participants who had mild (67/90 trials, 74%), mild to moderate (73/90 trials, 81%), and moderate (180/230 trials, 78.3%) dementia symptoms, and these participants found more of the prompts useful than did participants with severe dementia (33/90 trials, 37%).

**Figure 4:**
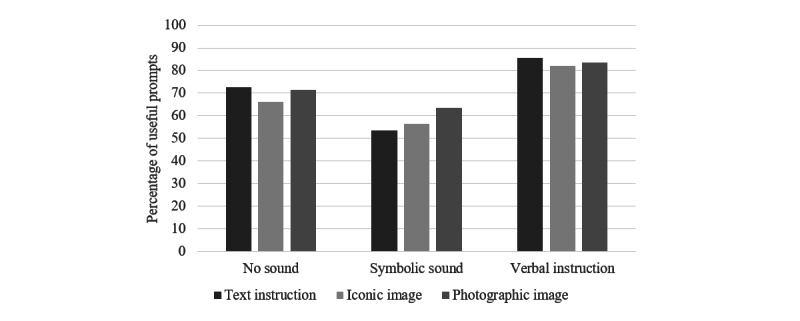
Percentage of useful prompts by prompt visual and auditory content.

Most importantly for our research questions, we examined what combinations of visual and verbal stimuli were the most effective in yielding useful prompts. Across tasks, the type of visual content made little difference to prompt usefulness. The most important prompt feature that impacted usefulness was the presence of the verbal instruction (compared to a symbolic sound or no sound). A chi-square analysis confirmed that the spoken verbal instruction was more useful than the other auditory conditions of a symbolic sound or no sound (*χ*^2^_2_=27.2; *P*<.001), whereas the different visual conditions (text instruction, iconic image, and photographic image) did not make a significant difference regarding prompt usefulness (*χ*^2^_2_=0.9; *P*=.65; Figure 4).

##### Prompt Success Across Time

We examined whether there was a difference in prompt success across trials to see whether useful prompts were more frequent on later trials (indicating learning) or early trials (indicating fatigue). To achieve this, we compared the average day number (1-9) and the average trial number (1-45) for useful versus not useful prompts. This analysis indicated no significant difference in average day or task number of useful versus not useful prompts (t_498_=0.85 and *P*=.20 vs t_498_=0.93 and *P*=.18, respectively). This suggests no evidence that people found the prompts more or less useful with practice and experience.

##### Combining All Predictors

Given that there was wide variation in the usefulness ratings across participants and that participants contributed multiple trials to the data set, we followed up these trial-based analyses with a stepwise (forward, likelihood ratio) logistic regression, with predictors added in order of participant, degree of dementia, task content, day number (1-9), trial number (1-45), auditory condition, and visual condition. Usefulness on each trial was the binary dependent variable. This analysis yielded a model with 4 significant predictors, confirming a significant effect of auditory condition (*B*=0.37; *P*=.004) over and above the significant contributions of participant (*B*=0.068; *P*<.001), dementia severity (*B*=−0.55; *P*<.001), and task content (*B*=0.21; *P*=.006). There was no significant contribution of visual condition (*B*=.26; *P*=.61). Overall, auditory prompt content predicted prompt usefulness over and above participant variance, but visual prompt content did not.

##### Time to Respond to Prompts

The time to respond to prompts was examined to understand which prompts elicited the quickest comprehension of information. In general, it took significantly longer for participants to select not useful (mean 30.70; SD 25.26 s) than useful (mean 19.19, SD 18.72 s; t_498_=5.62; *P*<.001). This may be due to care partners giving participants with dementia more time to consider the prompt before declaring that it was not useful. We examined whether auditory or visual content resulted in a faster response by conducting a 3 (auditory content)×3 (visual content) ANOVA on response times for the 353 trials in which the prompt was useful. This analysis yielded no main effects or interactions between auditory and visual content on response time (*F* values of <0.93 in all cases; *P*>.44 in all cases). Overall, auditory and visual prompt features did not impact time to respond to prompts.

##### Care Partner Support

**Figure 5:**
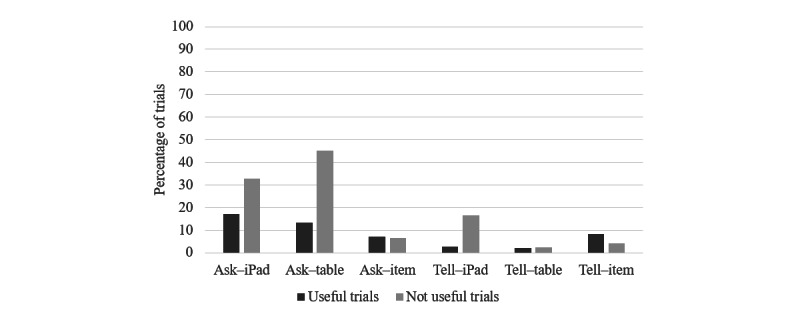
Percentage of trials in which support was given by support type.

To understand how much care partners were involved in helping participants with dementia engage with the prompts and complete the tasks, independent raters scored the video recordings captured by the iPad during each trial for the presence of care partners providing additional verbal support or instructions. Care partners were observed giving assistance by *asking for attention* when they asked the participant with dementia questions such as the following: “what is on the iPad?”; “what is on the table?”; and “what do you do with it?” Care partners were observed to provide assistance through *direct telling* when they told the participant with dementia what was on the iPad (eg, “it’s a toothbrush”), what was on the table (eg, “see the toothbrush on the table”), and what to do with the item (eg, “now brush your teeth”). Across the categories of support, *asking for attention* and *direct telling* were much more likely in trials rated as not useful compared to useful (chi-square values of >13.1 in all cases; *P*<.001 in all cases; Figure 5). Support in drawing attention was more frequent, whereby care partners scaffolded the participant with dementia by sensitively drawing attention if the prompts alone were not successful. The lower frequency of the direct telling form of support suggests that the content of the prompts was useful for participants with dementia and could be understood and acted upon once their attention was drawn to the task (Figure 5).

### Experiment 2

#### Overview

In experiment 1, we focused on simple tasks that could be completed with a single object by participants seated at a table. This approach enabled us to compare the specific visual and auditory features of the tasks while holding other aspects such as complexity constant. In experiment 2, we extended our findings to more complex multistep tasks that were a better match for ADLs, which are likely to involve a sequence of interactions with multiple objects. We wanted to determine whether people with dementia would be able to follow a multistep task through to completion with an appropriate sequence of prompts, noting that, in experiment 1, care partners were involved in maintaining participants’ attention to the task.

#### Methods

##### Participants

Participants were 9 people with dementia, all of whom were supported by a female care partner to complete the research. All participants in experiment 2 had previously completed experiment 1. Participants with dementia (4/9, 44% female; 5/9, 56% male) ranged in age from 58 to 82 years, with an average age of 74 (SD 7.43) years. Participants who were care partners (4/9, 44% spouses; 4/9, 44% adult children; 1/9, 11% siblings) ranged in age from 34 to 77 years, with an average age of 61 (SD 13.31) years. A total of 44% (4/9) of the participants with dementia spoke English as a first language, and 56% (5/9) spoke a language other than English. Participants had received a diagnosis of dementia between 9 months and 9 years before, with an average of 3.5 (SD 2.79) years. We did not formally record additional medical information regarding comorbidities beyond the participants’ dementia diagnosis, but participants were required to have sufficient visual and hearing ability to be able to see and hear the iPad prompts as part of our inclusion criteria.

##### Materials

###### Software for Prompt Delivery

As with experiment 1, software development was conducted to produce an iPad app for displaying prompts and collecting data. An iPad was issued to each participant, with the reliance on collaboration between researchers and care partners to facilitate data collection with participants with dementia in their home.

###### Prompting Conditions

To examine the best way to break down prompts for multistep tasks, we selected 2 common everyday tasks (“wash and dry hands” and “make a sandwich”) that were relevant for all participants and in which the same set of steps to complete the tasks could be used for all participants. Care partners confirmed the suitability of the 2 tasks before participation. Care partners were issued a detailed manual together with verbal phone instructions to assist with the standardization and delivery of the experimental tasks in the participants’ homes.

###### Prompt Content

**Figure 6:**
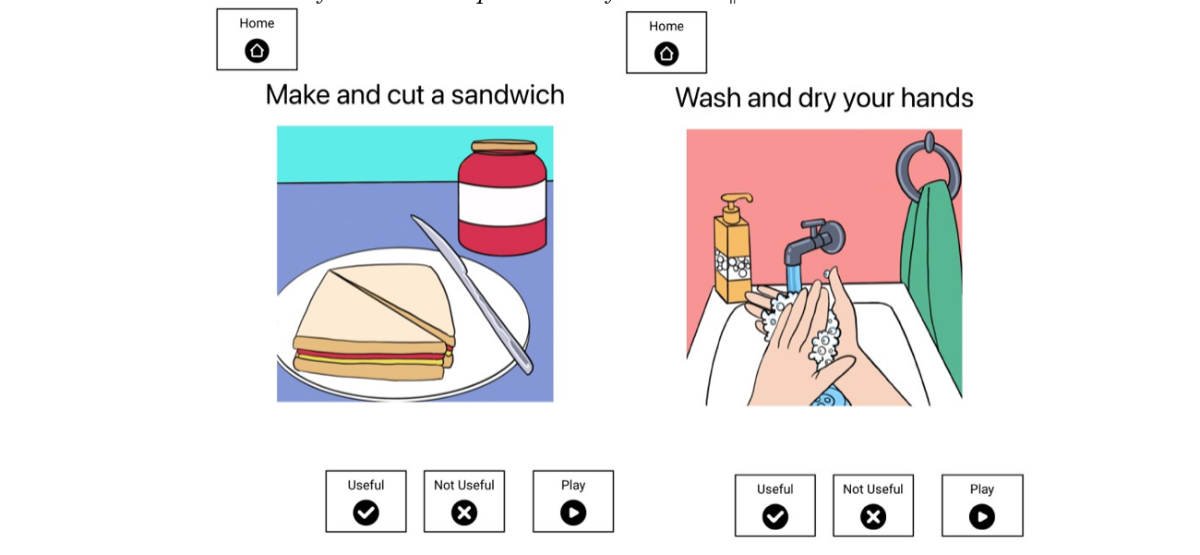
Visual stimuli for the 1-prompt version of the tasks.

To develop a uniform set of custom-designed icons for prompts, we worked with a professional graphic designer to create visual stimuli that were simple and clear, used high contrasting colors, and included all relevant components of the task in the image (Figure 6).

For each of the 2 tasks, we developed 1-prompt, 3-prompt, and 7-prompt versions (Table 2). Each visual image was combined with a text instruction and a verbal instruction. The verbal instructions were recorded by the research team in an Australian-accented female voice. Stimuli were designed to include images, text, and audible verbal instructions so that prompt content represented the most useful condition from experiment 1. The iPad automatically captured video and audio during the experimental sessions while the prompts were on the screen using the front-facing iPad camera.

**Table 2:** Prompt conditions (1, 3, and 7 prompts) for the 2 tasks.

Daily task	Prompt condition		
	1 prompt	3 prompts	7 prompts
Wash hands	Wash and dry your hands.	Wet your hands under the water. Wash your hands with soap. Dry your hands.	Turn on the tap. Wet your hands. Put soap on your hands. Wash your hands with soap. Rinse your hands. Dry your hands. Turn off the tap.
Make a sandwich	Make and cut a sandwich.	Get bread and fillings. Put the fillings in the sandwich. Cut the sandwich.	Put 2 pieces of bread on a plate. Butter 1 piece of bread. Choose your fillings. Put your fillings on the buttered bread. Put the second piece of bread on the top. Cut the sandwich. Put the sandwich on the plate.

##### Measures

###### Prompt Usefulness

For each prompt, care partners rated whether it was “useful” or “not useful” by selecting the appropriate button on the iPad.

###### Task Quality

To understand the quality of task completion in the different prompting conditions, care partners completed a task checklist during the experimental task and noted any relevant observations. The task checklist itemized all 7 steps involved in the 2 tasks, and on each trial, care partners checked which steps were performed by participants with dementia in response to the 1-, 3-, and 7-prompt versions. As such, care partners could score that a prompt was “useful” overall (via button pressing on the iPad) but could also identify when some steps of the task may have been omitted (eg, using soap), especially during the more condensed prompting conditions.

###### Care Partner Support

As in experiment 1, we scored the video recordings of each trial for care partner involvement. Independent coders scored the frequency and type of verbal assistance from care partners classified according to 2 domains (asking for attention or direct telling). All videos were scored for the presence of support in the 2 categories by 2 raters who were blind to the prompting condition. Agreement between raters was substantial (86.9%), and the ratings of the first rater were retained for analysis.

##### Procedure

###### Baseline and Setup

During an initial video call, participants were oriented as to the project. They provided informed consent and proxy consent where appropriate. If participants with dementia had not had a recent cognitive assessment with their health care provider, an MMSE [41] was conducted to assess the cognitive status of the participants with dementia and determine the consent pathway. Participants were given a detailed printed manual as well as verbal phone instructions about how to set up and conduct the tasks. Care partners were instructed that participants with dementia would be prompted to complete 1 multistep task each day, either washing their hands or making a sandwich. Care partners were asked to indicate the usefulness of each step by pressing “useful” or “not useful” depending on whether participants with dementia understood the prompt and completed the action. In addition, care partners were asked to complete a checklist each day to indicate the quality of activity completion in each prompting condition.

###### Experimental Phase

On each research participation day, care partners supported participants with dementia in working through 1 of the 2 activities in each of 3 prompting conditions for a total of 6 experimental trials. Conditions and tasks varied in a fixed random order so that, across the whole experiment, each of the 2 tasks appeared in each of the 3 conditions. For each activity, participants first viewed a screen that stated what activity was scheduled for the day and how many steps it would have. Once participants pressed “start,” the prompt sequence began. Care partners were asked to allow participants with dementia to respond to the prompt themselves without assistance. For each prompt, care partners rated whether it was *useful* (the participant with dementia completed or approximated the task) or *not useful* (the participant with dementia did not complete the task) by selecting the appropriate button on the iPad. Once rated, the next step appeared on the screen, with a final screen to indicate when the activity was completed. The iPad automatically captured video and audio during the experimental sessions while the prompts were on the screen using the front-facing iPad camera. Care partners used a hard-copy notebook to record the checklist of completed steps and any other observations regarding each day’s task.

###### Experience and Feedback Interview

After completion of the 6-day experiment, a semistructured interview was conducted with both the participants with dementia and the care partners together using the same core questions as those for experiment 1. The interviews were approximately 15 to 20 minutes in duration. Due to restrictions on face-to-face interactions with participants, all interviews were conducted via web-based videoconference and were recorded for transcription and analysis.

#### Results

##### Prompt Usefulness

To examine the benefit of breaking down an activity into multiple steps, we first examined the frequency of useful versus not useful care partner ratings in each of the conditions.

The 7-prompt sequence obtained the highest ratings of useful for both the sandwich and handwashing tasks. However, usefulness ratings were high across steps in all conditions (Table 3). We also noted that washing hands appeared to reach maximum effectiveness after 3 prompts, with no additional benefit gained from the 7-prompt version. Separate chi-square analyses confirmed no statistically significant difference in frequency of useful versus not useful ratings for steps in the 1-step, 3-step, and 7-step conditions in the 2 different tasks (*χ*^2^_2_=1.2 and *P*=.54 vs *χ*^2^_2_=1.5 and *P*=.48, respectively).

**Table 3:** Percentage of “useful” steps in the 3 prompt conditions (1, 3, and 7 prompts) for the 2 tasks.

Daily task	Prompt condition, n/N (%)		
	1 prompt	3 prompts	7 prompts
Wash hands	9/11 (82)	28/32 (88)	71/77 (92)
Make a sandwich	9/11 (82)	26/33 (79)	67/77 (87)

##### Prompt Usefulness and Dementia Stage

We examined any differences in prompt usefulness depending on stage of dementia. Data indicated a difference in the frequency of prompts being useful versus not useful depending on participants’ dementia severity (*χ*^2^_2_=26.8; *P*<.001). Participants with mild (80/88 steps, 91% useful) and moderate (102/109 steps, 93.6% useful) dementia had comparable results, whereas participants with more severe dementia found the prompts less useful (28/44 steps, 64% useful). In general, prompts in experiment 2 were more frequently rated as useful compared to those in experiment 1 regardless of participants’ dementia severity. This was perhaps because experiment 2 used the most effective prompting condition identified in experiment 1, combining a visual prompt with both an audible verbal instruction and a written text instruction.

##### Task Quality

The task checklist and care partner observations from each day were analyzed to understand the quality of task completion in the different prompting conditions and gain feedback on how we had chosen to break down the tasks into steps and whether there were steps missing. Across all participants, care partners indicated that most steps were performed in all prompting conditions. However, there were individual differences in care partners’ observations about which versions of the prompts were most effective. Some participants found that the 1-prompt condition was sufficient to support the participant with dementia in completing the activity. For example, some observations recorded in the 1-prompt condition included the following: “[participant with dementia] did it naturally, even rushed the steps,” “Washing hands is something my mother knows very well, so she is able to perform it very easily when prompted,” and “all instructions were performed.” However, some participants mentioned that the 1-prompt sequence did not include enough instructions. For example, observations in the 1-prompt condition included the following: “[The participant with dementia] left tap running on a drip, [it] was not turned off properly,” “I think the additional step ‘eat your sandwich’ may be beneficial as the sequence of steps does not state what to do with the sandwich once complete!” and “[participant with dementia] needed a prompt to find [the] hand towel.”

The 7-prompt sequence worked well for many participants. For example, an observation in the 7-prompt condition was as follows: “all of the above steps are easy to follow.” For some participants with dementia, the explicit instructions in the 7-prompt sequence encouraged them to stay focused on each step of the activity rather than rushing. For example, an observation in the 7-prompt condition was that the participant with dementia had “used more fillings” when making a sandwich compared to the 1- and 3-prompt conditions. Similarly, participants with dementia were observed by care partners to spend more time thoroughly washing their hands when following the 7-prompt sequence. However, for some participants with dementia, 7 prompts were too many. For example, observations in the 7-prompt condition were as follows: “At times...the 7 steps might have a few steps too many [as] mum was ahead of time doing the activity. She wanted to turn on [the] tap to rinse before the step was prompted” and “[participant with dementia]...pre-empts commands.”

The completed checklists and observations made by care partners highlighted the personalization of prompts necessary to benefit each person. They also helped to contextualize the useful and not useful ratings, reflecting different degrees of quality or thoroughness in task completion. Overall, findings suggest that, for some participants with dementia, more granular prompt breakdown facilitated more thorough completion of the ADL. However, for others, the additional prompts may have caused confusion. Ideal task breakdown may also vary for different tasks. For example, some tasks familiar to the person with dementia may only need a single-step prompt, whereas less familiar tasks may need more explicit and finely grained multistep prompts, suggested by the different patterns between handwashing and sandwich making.

##### Care Partner Support

**Figure 7:**
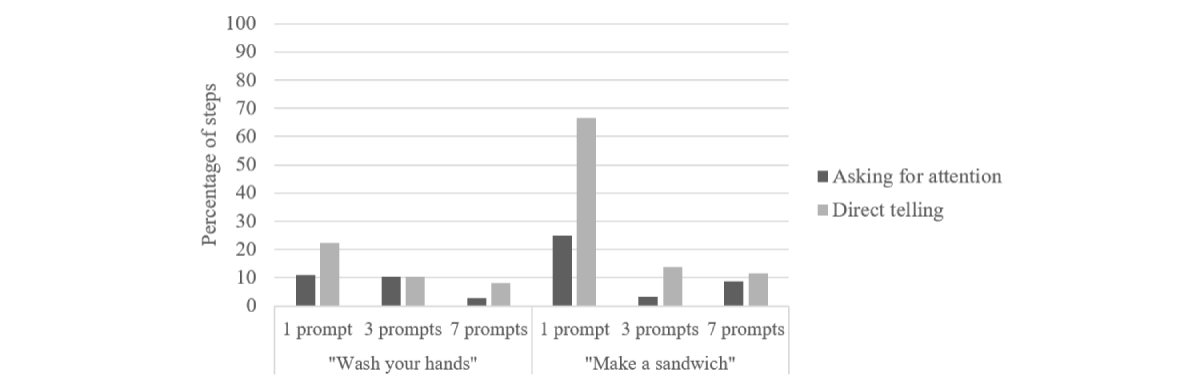
Percentage of steps in which care partners provided support by task type and condition.

Similar to experiment 1, we measured the extent to which care partners used verbal statements to help participants with dementia notice and understand the prompts and complete the prompted tasks. Statistical analysis indicated that the frequency of both “asking for attention” and “direct telling” was relatively higher when the prompt was unsuccessful than when it was successful, indicating that care partners provided support when they perceived that it was needed (*χ*^2^_1_=10.7 and *P*=.006 vs *χ*^2^_1_=10.1 and *P*=.004, respectively), as in experiment 1. Substantially higher levels of support were provided in the 1-prompt condition, particularly for the less familiar task of making a sandwich (Figure 7), and this mostly involved direct instruction, unlike in experiment 1.

To examine the impact of condition given the association with prompt success and that participants contributed multiple trials to the data set, we followed up these trial-based analyses with a stepwise (forward, likelihood ratio) logistic regression, with predictors added in order of participant number, prompt success, task content, and number of prompts (1, 3, or 7). The presence of care partner assistance in the form of “asking for attention” or “direct telling” on each trial was the binary dependent variable for the 2 separate regressions. For “asking for attention,” this analysis yielded a model with only 1 significant predictor, confirming prompt success as impacting the likelihood of drawing attention (*B*=1.71; *P*=.003). For “direct telling,” this analysis yielded a model with 2 significant predictors of prompt success (*B*=1.27; *P*=.008) and number of steps (*B*=0.21; *P*=.02). The negative relationships indicate that tasks with fewer steps had higher frequencies of direct telling over and above the effect of prompt success. This provides evidence that breaking tasks down into more granular steps increased the independence of people with dementia in using the prompting system.

### Experiment 3

#### Overview

Experiment 3 aimed to follow up our observation in experiments 1 and 2 that care partners frequently supported participants with dementia in responding to prompts by drawing their attention or directly telling them what to do, especially at the outset of the task to draw attention to the iPad, consistent with the challenges that people with dementia experience with task initiation. In experiment 3, we addressed two key questions about how to better support people with dementia in independently initiating and completing ADLs: (1) what kind of auditory alerts are most effective for gaining people with dementia’s attention so that they initiate a new task? (2) What kind of auditory alerts and what frequency are most effective for maintaining people with dementia’s attention to complete multistep tasks? While various kinds of alerts (audio and vibration) have been found to be effective in gaining the attention of people with an acquired brain injury [43,44], little is known about the potential role of alerts in supporting people with dementia to initiating and accurately completing everyday tasks [45].

#### Methods

##### Participants

A total of 17 people with dementia were recruited for this study, including 16 (94%) who participated with a care partner and 1 (6%) person with dementia who participated alone. Participants living with dementia (n=6, 35% female; n=11, 65% male) were aged 58 to 90 years, with a mean age of 76 (SD 8.76) years. Care partner participants (15/16, 94% female; 1/16, 6% male) were aged 25 to 88 years, with a mean age of 61 (SD 18.89) years. In total, 65% (11/17) of the participants with dementia spoke English as their native language; 35% (6/17) communicated most of the time in a language other than English. Time since diagnosis ranged from 3 months to 9 years, with an average of 3 (SD 2.30) years. Care partners were 56% (9/16) spouses, 38% (6/16) adult children, and 6% (1/16) formal carers of the participants with dementia. We did not record additional medical information regarding comorbidities beyond participants’ dementia diagnosis, but participants were required to have sufficient visual and hearing ability to be able to see and hear the iPad prompts as part of our inclusion criteria. The experiment ran over a 14-day period, and not all participants completed all trials. However, partially complete data were retained for analysis.

##### Materials

###### Software for Prompt Delivery

As previously, software development was conducted to produce an iPad app for prompt delivery and data collection, and an iPad was issued to each participant. Researchers and care partners collaborated to facilitate data collection with participants with dementia in their everyday home environment.

###### Coloring-In Activity

**Figure 8:**
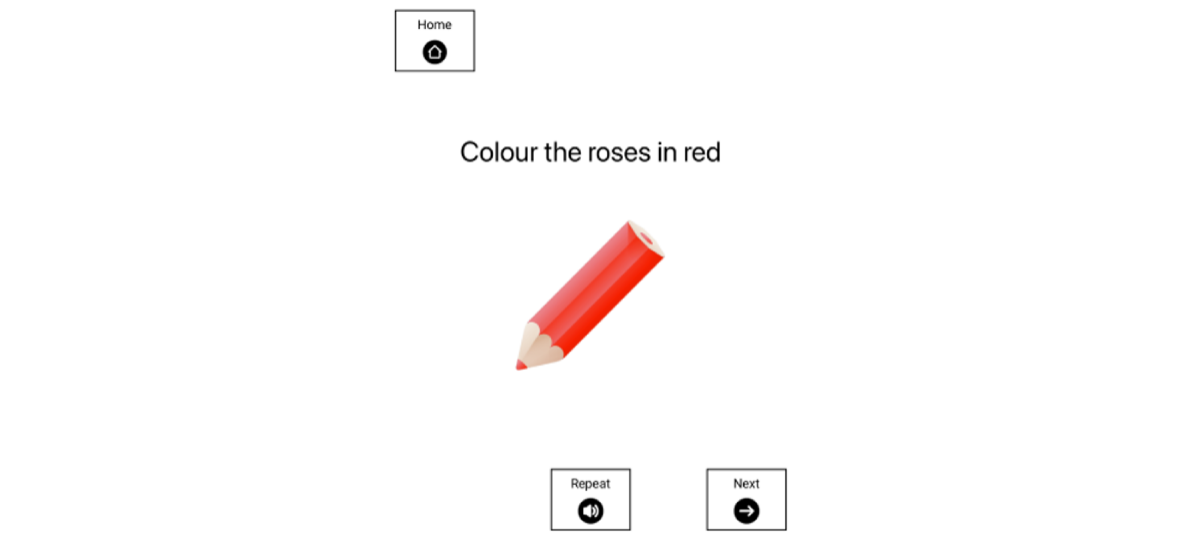
Example coloring prompt combining text, visual icon, and “Repeat” option for verbal instruction.

We designed a coloring-in activity to provide an experimental, multistep task that could be standardized across all participants. Our findings from experiment 2 emphasized the heterogeneity in the natural steps and sequence for completing ecological everyday tasks. Coloring pictures were selected from dementia-friendly coloring books, with a new picture provided each day in a physical printed booklet. We provided each participant with a coloring book and pencils with the same instructions and number of steps across all participants, enabling experimental control and an objective scoring system to determine task completeness and accuracy (Figure 8).

###### Prompt Content

The prompt for each of the 6 steps was multimodal based on the most effective conditions from experiments 1 and 2, combining a text instruction, verbal instruction, and visual icon of the colored pencil that the participant was instructed to use. Participants pressed *repeat* on the iPad if they wanted to hear the verbal instruction again. Participants pressed *next* on the iPad to receive the next step in the coloring activity (Figure 8).

###### Auditory Attention Alerts

A melodic tone called “Blackberry Spirit” was used for both the initiation and the attention maintenance alerts. This tone was selected based on evidence that receiving a soft tone notification is preferred for receiving noncritical messages than notifications with loud tones, which are deemed more intrusive [46]. We judged Blackberry Spirit to have a soft tone with a “happy” emotional association. Verbal instructions were recorded in an Australian-accented female voice for both initiation (“You have a new activity on iPad. Press ‘start’ to begin”) and maintenance (“When you have finished this step, press ‘next’ to continue”) alerts.

###### Care Partner Notebook

In experiment 3, we asked care partners to sit in the next room during the experimental sessions and allow the participant with dementia to navigate the task and the prompts by themselves. To facilitate this and score the usefulness of the different prompts, care partners received a notebook with a series of questions to answer on each day of the activity, including recording whether they had provided assistance with initiating or completing the task, rating the participant with dementia’s mood, indicating the other activities of the day, and recording any other observations or comments.

##### Procedure

###### Baseline and Setup

**Figure 9:**
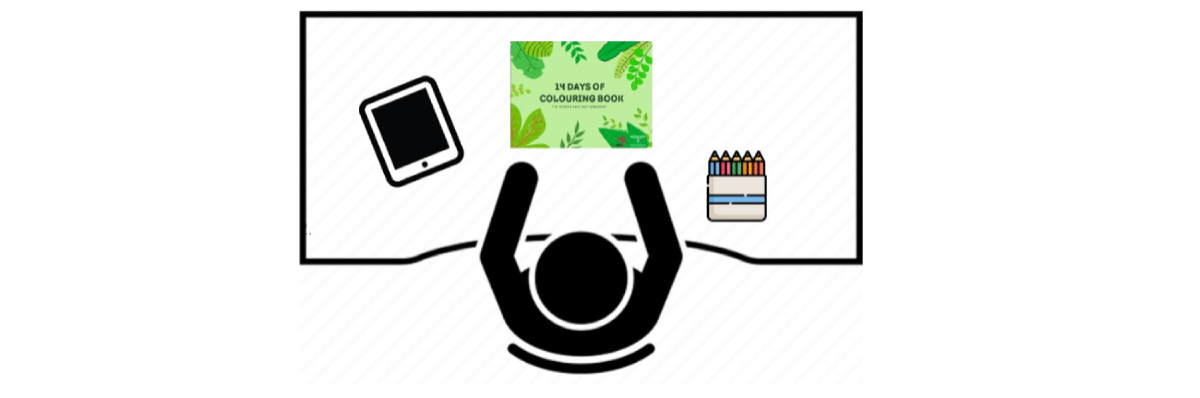
A visual representation of the at-home setup provided within the care partner manual, including positioning of the coloring task and iPad.

Care partners were given a detailed manual as well as verbal phone instructions regarding how to set up the at-home experimental task. Care partners were instructed to ensure that the participant with dementia was able to sit comfortably at a table, with the iPad, coloring-in book, and pencils within reach. The iPad was placed to the side of the participant with dementia to enable them to see and interact with it while being able to reach the objects on the table (Figure 9).

###### Task Initiation

At the beginning of each trial, an initiation alert played on the iPad. To test which initiation alert might be most useful for drawing the participant with dementia’s attention to the iPad task, we adopted a 2×2 within-subject design with Alert Type as the first independent variable. The initiator alert was either the Blackberry Spirit tone alone (tone-only condition) or Blackberry Spirit followed by the verbal instruction (tone+verbal condition). The second independent variable manipulated whether the iPad screen repeatedly flashed bright white while the initiation alert was playing to add a visual cue for gaining attention. Care partners were asked to allow the participant with dementia to notice and initiate the task themselves but, if they did not, to then direct them to commence the task for the day by drawing their attention to the iPad. Care partners recorded in their notebook whether they had provided assistance for task initiation.

###### Task Maintenance

Following task initiation, participants with dementia were presented with a sequence of 6 prompts to complete that day’s coloring-in activity. For each step, participants pressed *next* when it was complete and they wished to move to the next step. To examine whether alerts were useful for supporting participants with dementia to maintain focus and continue through the full sequence of steps in the activity, maintenance alerts were tested in a 2×3 experimental design varying 2 alert conditions (tone only vs tone+verbal) and 3 timing conditions (no alert vs 30-second interval vs 60-second interval). The resulting 6 conditions were presented in a fixed random order across days 2 to 13, with each condition appearing twice. Days 1 and 14 were always the no-alert condition to provide a baseline and a posttest of task completion. The iPad automatically captured video and audio during the experimental sessions while the prompts were on the screen using the front-facing iPad camera.

###### Experience and Feedback Interview

After completion of the 2-week experiment, a semistructured interview was conducted with both participants with dementia and the care partners together. Example questions included core questions from the previous experiments as well as some additional specific questions about the alerts, including the following: “Did the participant start the activity by themselves in response to the alert? Why or why not?” and “Did the alert during the task help the participant maintain focus?” The interviews lasted approximately 15 to 20 minutes. The interviews were conducted via web-based videoconference or in person and were audio recorded and transcribed for later analysis. Written transcripts were reviewed to identify common themes within and across participants.

#### Results

##### Task Initiation

We examined how often the participants initiated the task independently versus with assistance from their care partners by examining and coding the videos captured by the iPad and scoring the care partner reports recorded in their daily notebooks. Of the 210 initiation trials that had video data captured by the iPad, independent raters scored 91 (43.3%) trials as involving participants with dementia initiating the day’s task themselves and 119 (56.7%) trials as involving support from the care partner to commence the day’s activity. A total of 12.1% (29/239) of the presented trials were missing or could not be coded. Reports from care partners contrasted slightly with coded video results. Of the 204 trials with notebook entries, care partners reported that 129 (63.2%) trials involved participants with dementia initiating the day’s task themselves and 75 (36.8%) trials involved support from care partners to commence the day’s activity. Participants with dementia took between 1 and 299 seconds to initiate the task after the alert sounded. The average time taken to commence the task was 26 seconds. Taken together, these findings suggest that participants with dementia sometimes initiated the task themselves and sometimes relied on the care partner to do it for them. Coders also noted in the videos that participants with dementia were often seated at the iPad waiting for the task to commence and this may have influenced these findings.

To examine whether the nature of initiation alerts influenced the likelihood of trials being person with dementia initiated versus care partner initiated, we conducted 2 stepwise (forward, likelihood ratio) logistic regression analyses examining the impact of condition as well as accounting for participant-related variance. Predictors were added in order of participant, participant’s dementia category, experimental day (1-14), alert type (tone only or tone+verbal), and screen flash (present or absent). Whether the task was initiated by the participant with dementia or by the care partner was the binary dependent variable for the 2 separate regressions, one using the independent video coding and the second using the care partner ratings of who initiated the task. A significant model emerged (*χ*^2^_1_=28.3; *P*<.001; *R*^2^=0.169), with only 1 significant predictor of dementia category (*B*=−0.751; *P*<.001). These results suggested that the auditory and visual nature of the initiation alert had no impact on the likelihood of the participant with dementia initiating the activity independently and that people with more severe dementia were less likely to independently initiate the task. Both tone-only and tone+verbal alerts were similar in effectiveness, with no advantage of adding a voice to the tone and no impact of the additional visual cue of the flashing screen. Overall, initiation alerts were moderately effective, with participants with dementia independently initiating the task 43% to 63% of the time regardless of auditory and visual condition.

##### Task Maintenance and Completion

To assess the value of attention maintenance alerts, we scored participant performance on the coloring task by examining the coloring books and assigning each step on each task a score for *accuracy* (whether the correct color was used) and *completeness* (score from 1 to 5 indicating degree of coverage). Participants with dementia used the correct color on 78.97% (1093/1384) of the steps and the incorrect color on the remaining 21.02% (291/1384) of the steps. Participants received an average completeness score of 3.85 (SD 1.67), indicating 60% to 80% coverage on each step. However, the modal score was 5 (>80% completeness), which was achieved on 57.08% (790/1384) of the steps. Overall, these data suggest that participants with dementia could follow the prompts and complete the coloring task successfully and accurately, although their completion and accuracy varied across individuals and days. Participants with more severe dementia had lower accuracy and completeness scores than those with milder dementia. The average coloring step lasted for 378 (SD 297.65) seconds (just over 5 minutes), although this varied across participants and days.

Most importantly for our research questions, we examined whether the likelihood of participants with dementia pressing *next* independently, accuracy, completion, and time spent on each coloring step varied systematically as a function of the content and timing of maintenance alerts. We conducted 3 stepwise (forward, likelihood ratio) logistic regressions, with predictors added in order of participant number, dementia category, prompt presence (present or absent), prompt content (none, tone, or tone+verbal), and timing (none, 30 seconds, and 60 seconds) Binary dependent variables were whether participants with dementia pressed *next* independently (yes or no), accurate color choice (yes or no), and completeness (>80% or <80%) for 3 separate regressions. A fourth linear regression was conducted with the same predictors for the continuous dependent variable of time taken on each step (Table 4). Across dependent variables in all 3 logistic regressions, the only significant predictors were participant ID and dementia category. Overall, there were individual differences in independence and task completion, but these were associated with individual differences and task familiarity rather than with the presence, content, or timing of the maintenance alerts. The only exception was timing. The regression yielded alert type as a positive predictor of time spent on each step, indicating an advantage for sound+verbal trials, as well as alert trigger as a negative predictor, indicating an advantage for alerts at shorter intervals.

To better understand the impact of alert type and alert trigger on the time spent on each step, we compared mean time spent on each coloring step across conditions. A 1-way ANOVA across the 3 alert type conditions (none vs sound only vs sound+verbal) indicated no significant effect (*F*_2, 880_=1.40; *P*=.25). A 1-way ANOVA across the 3 alert timing conditions (none vs 30 seconds vs 60 seconds) indicated a significant effect of timing (*F*_2, 880_=9.43; *P*<.001). Follow-up comparisons indicated that participants with dementia spent significantly longer on steps that had maintenance alerts at 30-second intervals (mean 453.31 seconds; SE 15.36) than on steps that had no alerts (mean 371.18 seconds; SE 21.49; *P*=.006) and alerts at 60-second intervals (mean 364.35 seconds; SE 15.84; *P*<.001), with no significant difference between 60-second alerts and no alerts.

Overall, the findings from experiment 3 supported that people with dementia could accurately complete a sequence of multiple task steps supported by audiovisual prompts delivered on the iPad to both initiate and persist through a sequence of steps. Although there was some heterogeneity in whether people with dementia initiated the task independently and operated the iPad to move through the steps independently, we did not find clear evidence that the nature of auditory initiation or maintenance alerts made a difference regarding independent engagement with the prompts. Instead, individual differences, and particularly dementia severity, as well as experience with the task over the course of the experiment had a bigger impact. These findings suggest the value of iPad-delivered prompts as people with dementia could engage with them and use them to complete a sustained activity, with high rates of accuracy and completeness. We did find some evidence that maintenance alerts at 30-second intervals might help people with dementia persist with a task for longer. However, as people with dementia were supported to initiate the tasks approximately half the time regardless of alert condition, future work is needed to test alternative ways of initiating and maintaining attention if independent use of a prompting system without care partner support is a valued goal.

**Table 4:** Regression analyses predicting independent use, color accuracy, color completeness, and time spent on each step.

Dependent variable	Significant predictors	Model chi-square (*df*)	Model *R*	Model *R*^2^	*P* value	Correctness (%)
Independent initiation	Participant ID, dementia (negative), and day (positive)	123.65 (3)	—^a^	0.185	<.001	66.4
Color accuracy	Participant ID, dementia (negative), day (positive), and step (negative)	85.54 (4)	—	0.134	<.001	80.8
Color completeness	Participant ID, dementia (negative), day (positive), and step (negative)	139.46 (4)	—	0.180	<.001	66
Time spent on each step	Day (positive), step (positive), alert type (positive), and alert trigger (negative)	—	0.235	0.055	<.001	—

^a^Not applicable.

#### A Synthesis of Experiences and Feedback Across all 3 Experiments

##### Overview

In all 3 experiments, we conducted detailed interviews with care partners alongside participants with dementia to discuss their experiences of the prompts and the tasks. We explored what participants found most useful about the prompting system and what features they would like to see in future assistive technology devices. As the questions and resulting themes were similar across all experiments, we present a synthesis of the interview findings in the following sections. These insights nuance our experimental findings and inform key recommendations for future assistive technology development.

##### What Aspects Did Participants Find Useful and Not Useful?

Care partners provided mixed reports on the usefulness of the prompting system. Most carers found the prompts to be useful to at least some degree. However, the perceived usefulness of the system varied according to the participants’ dementia severity, consistent with our behavioral data. Participants in the early (mild) stages of dementia were less likely to report difficulties following the prompts and were able to complete the task independently. Care partners reported variability in participant mood and, therefore, the usefulness of the prompting from day to day (eg, “There are days he [enjoyed it]. There are days he [got] frustrated” [experiment 3]). Overall, findings highlighted the importance of customizability and personalization of the system to manage variability in preferences and abilities of people with dementia both across individuals and across time. Despite this variability, there were common themes regarding the benefits and challenges associated with the different prompts tested across the 3 experiments, which are detailed in the following sections along with care partner suggestions for further development and uses of a prompting system.

###### Useful Features

Care partner participants reported that the prompts were useful and practical and they generally liked the design of the visual pictures and icons (eg, “nice, simple pictures” [experiment 1]). Concerning auditory prompts (verbal instructions and symbolic sounds) in experiment 1, care partners reflected that the verbal instructions were more useful than the symbolic sounds as the association between the target object and action and the symbolic sound was more abstract, consistent with behavioral data. The verbal instructions were also perceived as helpful by care partners in later experiments:

The [verbal] instructions did [motivate him]. Yes, he would wait to hear “Colour the lady’s hair green” or whatever. And then that was like “Oh, I have a task,” and then he would do it.

Experiment 3

In experiments 2 and 3, some care partners found that the person with dementia’s ability to complete the multistep tasks and associated activity improved over time and with repetition. This effect encouraged these care partners to continue with the activities at home. For example, as a result of watching her husband make a sandwich independently with prompts, one care partner (experiment 2) reported an intention to continue supporting him with prompts to prepare his own lunch instead of making it for him as she had done previously. Similarly, in interviews following experiment 3, some care partners reported that the person with dementia enjoyed the coloring in and they were continuing the activity at home:

Yeah and [Dad’s] still doing it actually.... Now, when his carers come, they draw things or do writing, and he colours it in. So it started something that’s entertaining for him. So that was very useful.

Experiment 3

In this way, participating in the research led to a focus on retained abilities and benefited day-to-day quality of life. It also demonstrated the potential for a prompting system to connect people with dementia with meaningful leisure activities in addition to the completion of ADL tasks.

In experiment 2, care partners observed that the granular nature of the prompts for the multistep tasks resulted in a more thorough completion of the ADL task, improving task quality in a way that was not always captured by our binary scoring of success. For example, several care partners reported that, with the support of the prompting system, the participant with dementia had used soap when washing their hands, a step usually omitted when not prompted, or had washed their hands more thoroughly. In addition, across experiments, participants reported that they appreciated the simple app interface and the ease of pressing the “home” button to return to the start. They also reported the value of having a dedicated device that only contained the prompting program (“without all that other stuff on it”) to increase the simplicity of interacting with it.

###### Challenges

Some care partners reported that the need to locate buttons on the iPad was a mismatch with their family member’s abilities due to their stage of dementia (eg, “So [the verbal instructions] part did motivate him. [but], pushing buttons and messages on the screen—no” [experiment 3]). Some care partners mentioned that the participant with dementia would have more success completing even simpler tasks if multiple, more granular prompts were given. For example, at times, even simple 1-step tasks needed multiple prompts to complete (eg, “He knows where the remote is, but has difficulty finding the right button to press” [experiment 1]). Similarly, in experiment 3, a care partner reported the following:

[The task] “colouring the leaves green” involves both finding the right colour pencil and identifying the correct part of the picture.

This feedback suggests that the “right number” and granularity of steps may vary depending on the individual and the degree of support they need for a particular task.

Care partner responses indicated that the usefulness of the prompts was impacted by how well the prompt content matched an individual’s experiences and preferences. For instance, one care partner in experiment 1 commented that the generic picture of the television was too old-fashioned and, therefore, was hard to recognize as referring to the participant with dementia’s own television. These complexities highlight the need to personalize prompt content and structure to ensure that it matches the needs and experiences of the individual user.

##### Independent Use and Initiation by People With Dementia

Care partners of people with dementia in later stages generally expressed doubt about whether they would initiate and use a prompting device on their own:

I think maybe for somebody with very early dementia it might [be] useful, but I think for my mother’s stage it’s not so useful...it becomes just noise telling her to do something, and she’s not able to grasp [what to do].

Experiment 3

An overarching theme from carer feedback across the experiments, consistent with our behavioral data, was that care partners felt the need to be involved in directing the attention of people with dementia to the iPad prompts. For example, carers noted that their presence was needed to provide repetitions of the prompt, break the prompt down into smaller steps, or focus the participant with dementia’s attention. They also reported that participant with dementia found the initiation of the activity difficult and their ability to complete it was improved with scaffolding and support from the care partner. For example, some reported that screen-based prompts or activities were unsuccessful in capturing the attention of the participant with dementia, and others were unsure of whether the participant with dementia would understand the prompts and content on their own:

So it was that motivation to get started, and so I’m just not sure how it would work in practice. If she was alone, using the iPad, whether she would actually turn it on or not, I don’t know.

Experiment 1

Despite these concerns, some care partners were more optimistic about people with dementia being able to use a home assistant device through strategies such as developing a routine of checking a device in the morning to orient themselves for the day or learning to use the prompts independently over time with practice and familiarity (eg, “It might take a few goes, but she would probably be able to do it on her own” and “by the end, she was able to do it much faster; at the beginning, where she was unsure she would look to me” [experiment 1]). Others also discussed whether the participant with dementia would advance to the next step without the care partner there to press the button and suggested considering a timed system that automatically advanced to the next prompt to reduce the demands. Participants with dementia also reflected on the anxiety of getting things wrong and a worry that the “machine” would break or freeze if they pressed the wrong thing. However, care partners observed that participants with dementia became less anxious and more accomplished with using the prompts with practice and repetition and enjoyed the opportunity to interact with technology. Although we were concerned with the time demands of the repeated experimental sessions, some participants expressed that the experiments were too short and the person with dementia was just becoming familiar and comfortable with the tasks at the point in which they ended:

Because at the beginning she’s a bit nervous, so it’s about getting used to the technology and it’s hard to judge how well she’s doing via the prompts. So, it’s almost like, [the] last couple of days, where she’s starting to get it, especially some of the similar ones that keep coming up like brushing your teeth, drinking water, the ones she could become familiar with. Towards the end the minute she sees it she knows what it is.

Experiment 1

###### Auditory Alerts as Attention Grabbers

Given that there was a great deal of variation in whether participants with dementia initiated the task and that it did not depend on the nature of the initiation alert in experiment 3, we sought to understand the reasons for this variation by asking care partners during interviews about whether the alerts were effective at grabbing attention for the participant with dementia. Interviews with care partners identified that a common reason given for participants with dementia not initiating the task was confusion related to understanding and interpreting the alerts and prompts:

When it first started...when it’s buzzed...she knows something’s buzzing...so she knows she needs to get started but then I think the first step she tries, and then I think she gets caught up and not know what to do. And also, like, I think it’s a bit complicated for her in terms of that she doesn’t know where to press, whether it’s pressing on the colouring book or pressing on the iPad.

Experiment 3

But no, those alerts be it on the iPad or on the handheld, did not work [for mum]. It just didn’t work.

Experiment 3

##### Suggestions for Future Device Development

Across experiments, care partners were given the opportunity to suggest improvements to the prompts and prompting system. Suggestions included ways of improving the hardware and user interface, such as the use of a larger device, potentially mounted or fixed in place so that it could not get lost or misplaced. They also suggested making the attention-grabbing prompts deliverable on a more portable device rather than requiring users to be in front of the iPad or noticing a new task from across the room. For example, several care partners suggested incorporating a wearable to bring the participant with dementia’s attention to the task even if they were not in front of the iPad or so that reminders could be provided in nonhome locations (eg, taking medication while out at a friend’s house). Such a wearable would be too small for the screen display to be useful but could emit voice instructions. One care partner participant suggested that videos or active prompts showing the movement and the action involved in a task would be helpful for people with dementia to copy compared to the static visual images that we tested. Finally, participants mentioned that the device could be voice activated rather than requiring a button press on the screen to interact with it and progress through tasks.

##### Suggested ADL Tasks for Prompting

On the basis of their experience of the prompting system, we asked care partners to reflect on what ADL tasks could be potentially supported using home prompting devices. Although we received a range of suggestions, common responses included health and self-care activities such as taking medication, drinking water, and eating; hygiene activities such as taking a shower and brushing teeth; and well-being and recreation, such as doing some exercise, listening to music, and calling a friend or family member. Care partners found our approach of breaking tasks into substeps useful and suggested that additional ADL tasks could be broken down and prompted in similar ways. Care partners also suggested having options to display (and schedule) set tasks for some days and not others or to orient people with dementia with a list of reminders each morning (eg, “today we are doing...”). Care partners also noted that even short periods within the day (eg, “20 minutes”) while the person with dementia independently showers or shaves would be a valued source of independence and respite for both parties.

### General Discussion

#### Principal Findings

We aimed to compare the usefulness of different features of tablet-based prompts to support people with dementia in completing everyday ADLs at home. Across 3 experiments, we found that people with dementia could engage with tablet-based prompts and use them to complete activities. Prompts were more useful for people with mild and moderate dementia compared to those with severe dementia, but even participants with severe dementia could understand and respond to prompts on most trials, particularly when prompts combined auditory and visual modalities. In experiment 1, we found that the most effective prompts included an auditory verbal instruction regardless of their visual content. In experiment 2, we found that breaking complex tasks into substeps could support task completion as well as task quality and independence. Although the ideal granularity of steps depended on the individual and the task, people with dementia were observed to need less support when there was more than a single prompt. In experiment 3, we found some benefit of auditory prompts for drawing and maintaining attention but no difference between a tone alone and a tone combined with a verbal call to action. Combined with feedback from care partners, these findings suggest that fully independent use of prompting technology may be a challenging goal and that different hardware choices beyond a touch-screen tablet may be better suited to this user group to facilitate attention and engagement. Regardless, a degree of independence and engagement supported by the prompting system was valued by both people with dementia and care partners even if care partners were involved at times in facilitating its use.

#### Prompt Modality

Our experimental research comparing different kinds of visual and verbal stimuli to each other found that the presence of an audible verbal instruction was associated with task success. The nature of the visual content, whether text, icon, or photo, did not appear to influence prompt usefulness over and above the presence of an auditory verbal instruction. This was consistent with care partner observations and feedback as well as with other studies that combined auditory and visual components when designing prompts, suggesting the particular effectiveness of direct verbal and text-based prompts [19]. Direct instruction, provided both verbally and visually via text, appeared to be the most effective, removing ambiguity about what the task was.

There was also evidence that prompting was more effective for people in early to moderate stages of dementia, with less success for those whose dementia was more severe, although even those with severe dementia could respond to the prompts at least half the time. However, gaining experience and familiarity with a prompting device during the earlier stages of dementia may enable people to continue using it as their dementia progresses. We did not find a learning effect in experiment 1, but regression analyses in experiment 3 suggested that the accuracy and completion of the coloring task increased across the 14 days. Participants also reported that they appreciated the prolonged experimental period that allowed people with dementia to overcome their initial anxiety and gain confidence and familiarity with the tasks. This is consistent with other research showing that those who were more successful responding to a prompting device used it for longer, over more days, and on more occasions [24], emphasizing the role of confidence building and successful use in motivating further use. A longer period of regular use may have even more advantages and may have boosted performance for those with more severe dementia, but future research is needed to examine this question.

#### Prompts for Complex Tasks

Generally, increasing the number of substeps within a task increased the success of the prompts and the likelihood of task completion and reduced the extent to which care partner support was needed. Observations indicated that the ideal breakdown varied according to the individual and their degree of cognitive impairment as well as with the task complexity and familiarity. These factors are likely to combine to make the optimal breakdown of steps idiosyncratic to the individual such that the ability to tailor or select and deselect substeps may be ideal for prompt usefulness. We did not identify any previous research examining the optimal granularity for breaking down tasks when providing prompts to people with dementia, although some of the prompting devices reported in previous literature did break tasks down into substeps. For instance, the COACH handwashing system provided 5 steps for washing hands [47]. We found that breaking tasks down into multiple steps could be critical for ensuring their completion and increased independence. For instance, one participant could not wash his hands independently because he would forget to turn off the tap, and others would neglect to use soap if not reminded. In cases such as these, single prompts may be insufficient or even counterproductive for task completion. However, the optimal granularity and the specific step content varied depending on the individual and the specific task, making personalization essential for success [24,48,49].

#### Grabbing and Maintaining Attention

Our analysis of the videos indicated that care partners were often involved in drawing the attention of people with dementia to the task and ensuring task completion. Moreover, although we trialed different methods for the system to gain and maintain attention, we did not identify a particular type of alert that made independent initiation and task maintenance more likely than another as the tone alone and the tone combined with a verbal call to action had similar outcomes. On the basis of feedback, auditory alerts were reported to be difficult to interpret for people with dementia even when accompanied by a verbal reminder. Future research is needed to test other ways to support people with dementia to notice and initiate the task and persist through the steps of complex ADLs. One suggestion from care partners was the inclusion of an associated wearable device to tell people with dementia when there was a new task. Other suggestions included using the person with dementia’s name in the verbal call to action to gain and maintain attention across multiple steps, which was the approach adopted in a previous study [47]. Another study used a Bluetooth earpiece to directly provide verbal prompts to people with dementia wherever they were without relying on them being close to a device; however, this approach may not be suitable for all people with dementia [21]. Further research is needed to determine how to support people with dementia to independently notice and initiate a task when prompted by a home-based system. However, we noted that care partners and people with dementia still valued the partial independence provided by the prompting system even when care partners scaffolded its use.

#### User Experiences and Avenues for Future Development

Generally, care partners were positive about the prompting system and the characteristics and content of the prompts. They had a range of specific feedback, some of which was idiosyncratic to the individual participant. Feedback that generalized across participants was the need for a custom, dedicated device, potentially including a wearable to enhance portability. Consistent with our analysis, care partners reported that success at the different tasks and usefulness of the prompts was impacted by how well the prompt matched an individual’s experiences and preferences. Care partners reflected lack of certainty regarding whether such a system could be used independently, but both care partners and people with dementia valued the potential for independent use.

There was a range of specific recommendations that should be incorporated into future device development. This included the need for large display screens and large buttons. In addition, buttons should be clearly marked and kept to a minimum, and voice control may be more successful. Care partners suggested that the system may need multiple devices or screens in different rooms so that they could be in fixed, predictable locations and “plugged in” rather than needing to be charged. In addition, the system could be customized to deliver prompts automatically at set times. A recent review also noted the potential benefit of time-based prompts, especially as dementia progressed, but noted that the timing of transitions would need to be tailored and calibrated for the individual [11]. Overall, engagement remains a key challenge, particularly with remembering to use an assistive device and maintain attention to multiple steps in a sequence.

A major emerging consideration regards customizability and tailoring built into the prompting system [24,48]. We noted that much of the feedback about the usefulness of specific steps or prompts was idiosyncratic to the individual, their capacity, the specific task, and the home environment. It was challenging to generalize regarding the prompt content provided for people with dementia, and it varied depending on the individual and the task. Small mismatches between needs and expectations of the user and how the device behaves can render a system useless by creating confusion or uncertainty. Future development could focus on a technological solution that allows care partners or people with dementia to choose or edit the tasks and the steps that they want displayed to them (as in the study by Harris et al [24]). We also noted the wide range of content that care partners and people with dementia were interested in having supported through prompts. This included a focus on instrumental ADLs but also a focus on more meaningful activities, such as music listening, and facilitating social connection and reminiscing. Although much assistive technology development has focused on ADLs and safety [50], richer, meaningful activities are crucial for promoting positive quality of life for people with dementia and supporting relationships between people with dementia and care partners, and these should be considered in the development of prompting technology.

#### Limitations and Future Directions

Our research had a range of strengths, seeking generalizability by examining a set of prompts that were fixed and standardized across participants to examine how prompt features impacted their usefulness. However, this necessarily meant that the visual and auditory content of the prompts was generic rather than personalized. Although we made attempts to select the optimal content based on pilot-testing, it may have provided a poor match for some individuals, and this may have particularly disadvantaged the visual prompting condition in experiment 1. Future research is needed to examine whether there is more benefit from the visual prompt content when it is personalized to the individual, matching their own environment (ie, depicting their own television or their own bathroom sink) instead of being generic. Relatedly, we relied on care partners to indicate prompt usefulness. This meant that the research could be conducted in an ecological, everyday home setting over a long testing period rather than requiring the presence of researchers. However, carers may have been biased to support “good” performance and indicate that a prompt was useful when it was not. We deliberately framed our research questions regarding the usefulness of the prompts rather than the performance of people with dementia to reduce this bias, and we emphasized to participants that we wanted to know about prompts that were not useful just as much as those that were. However, future research could consider other ways of recording the outcomes of prompting, especially as pandemic-related restrictions on face-to-face testing have eased. Finally, we did not record detailed information about participants’ comorbidities, including limitations to their mobility, vision, or hearing. These individual differences are likely to shape needs and what kind of prompting is best suited to whom, and this would be a fruitful avenue for future research.

#### Conclusions and Recommendations

Overall, our findings suggest promise for future development of assistive technology to support people with dementia and their care partners. Participants with dementia could become familiar with and use a touch screen–based prompting device to complete a range of activities, especially when prompted with a combination of verbal and visual information and when activities were broken down into more granular substeps. We found that participants with dementia responded to prompts more than half the time across experiments, including people with more severe cognitive impairment when prompts combined visual content with direct verbal instructions. Therefore, we recommend that future prompting technologies include both a direct verbal instruction alongside visual prompts to maximize task success and that they enable complex tasks to be broken down into customizable substeps. Care partners were often involved in scaffolding task initiation and drawing the attention of people with dementia to ensure task completion, and additional attentional alerts did not appear to influence this. Overall, people with dementia and their care partners are interested in assistive technology that is intuitive and able to be personalized for the individual to make it work for their needs and context, prompt valued tasks, promote independence and self-esteem, and enhance quality of life. Our findings highlight the value of working meaningfully with intended users within their everyday settings to understand the reasons why assistive technology is experienced as useful or not useful as well as the need to move away from one-size-fits-all solutions to support people with dementia.

## Abbreviations

ADL: activity of daily living
COACH: Cognitive Orthosis for Assisting Activities in the Home
MMSE: Mini-Mental State Examination
